# Point-of-care serology in feline leishmaniosis: A diagnostic gap requiring species-specific solutions

**DOI:** 10.1016/j.crpvbd.2026.100448

**Published:** 2026-10-10

**Authors:** Sergio Villanueva-Saz, Diana Marteles-Aragüés

**Affiliations:** a Animal Pathology Department, Veterinary Faculty and Instituto Agroalimentario de Aragón – IA2, Universidad de Zaragoza-CITA, Miguel Servet 177, Zaragoza, 50013, Spain; b Clinical Immunology Laboratory, Veterinary Faculty, University of Zaragoza, Zaragoza, 50013, Spain

**Keywords:** Cat, Diagnosis, Feline leishmaniosis, Immunochromatography, *Leishmania infantum*, Serology

## Abstract

Feline leishmaniosis (FeL) caused by *Leishmania infantum* is increasingly recognised as a clinically and epidemiologically relevant condition in endemic regions. However, unlike canine leishmaniosis, rapid point-of-care serological testing has not become an established component of routine feline diagnostic workflows. This gap is important because feline immune responses to *L. infantum* differ from those observed in dogs, while the performance of serological methods varies substantially according to assay format and host-specific characteristics. We argue that FeL requires species-adapted point-of-care serology rather than direct extrapolation of canine diagnostic platforms. Such assays should not be considered stand-alone tools for confirming clinical disease, but rather first-line triage tests integrated into a multimodal diagnostic pathway. Their validation should include well-characterised clinical populations, assessment of low-level antibody responses, cross-reactivity and reproducibility, and methodologies capable of addressing the absence of a perfect diagnostic gold standard. The principal unmet need is therefore not simply the availability of another rapid test, but the development and rigorous validation of feline-specific assays that can improve early clinical recognition and appropriately guide confirmatory investigation.

## Highlights

• Feline leishmaniosis still lacks validated point-of-care serology.
• Canine serological paradigms should not be directly extrapolated to cats.
• Feline-adapted ICTs may improve first-line clinical decision-making.
• Rapid serology should support, not replace, confirmatory diagnosis.
• Species-specific ICT validation should be a diagnostic priority.

## Graphical abstract

**Figure:**
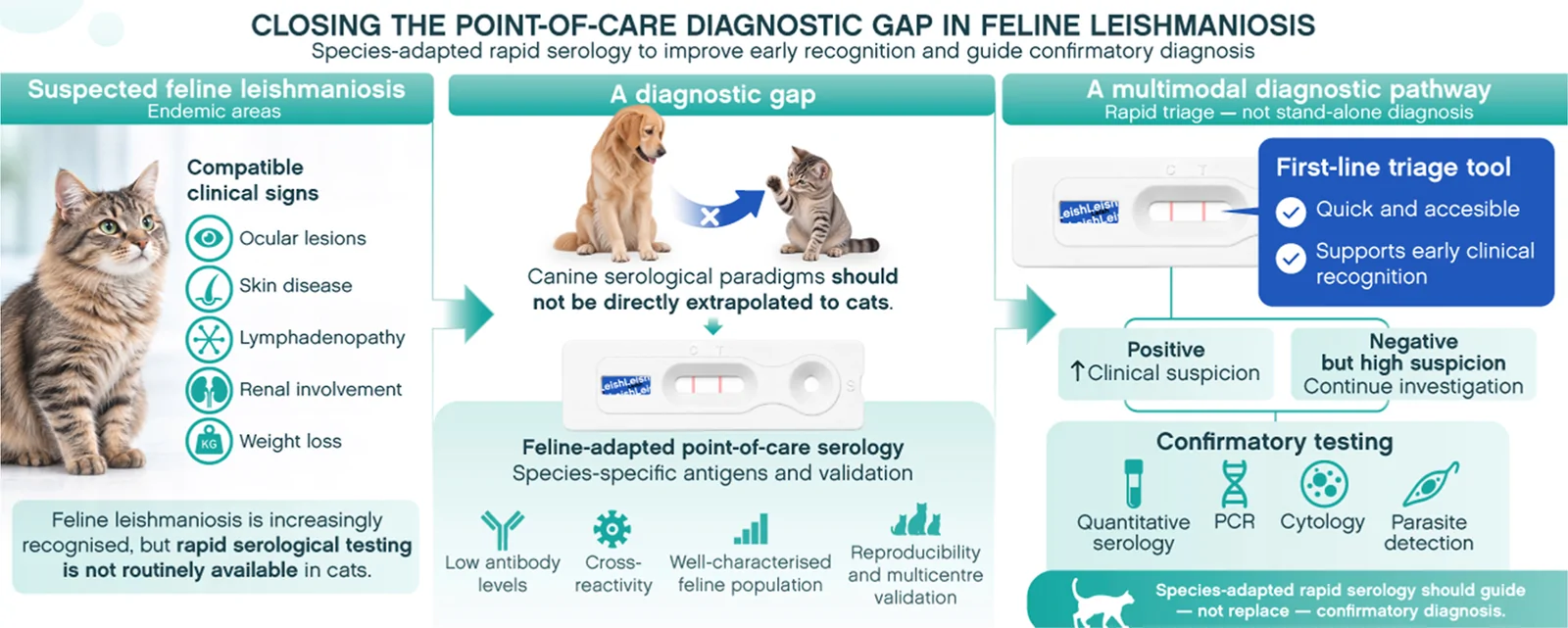

## 1 Diagnostic gap

Feline leishmaniosis (FeL) is increasingly recognised as a clinically relevant disease in *Leishmania infantum*-endemic areas, particularly in the Mediterranean Basin (Pereira and Maia, 2021; Garcia-Torres et al., 2022; Carbonara et al., 2024). In addition, accumulating epidemiological and xenodiagnostic evidence indicates that infected cats can contribute to the transmission cycle by infecting competent phlebotomine sand-fly vectors (Maroli et al., 2007), although their population-level importance as reservoirs remains less clearly established than that of dogs. The 2015 LeishVet recommendations therefore emphasised that FeL should be considered in cats presenting with compatible dermatological, ocular, oral, lymph node or systemic abnormalities, particularly in endemic areas (Pennisi et al., 2015). More than a decade after publication of these recommendations, however, an important diagnostic gap persists: the limited availability of rapid point-of-care serological assays specifically developed and validated for cats. This limitation is explicitly acknowledged in the updated LeishVet recommendations, which note that no commercial serological tests for feline *Leishmania* infection have yet been externally validated and published in a peer-reviewed journal, and caution against assuming that rapid tests developed for dogs are diagnostically accurate in cats (Pennisi et al., 2026).

Recent epidemiological evidence indicates that feline exposure to *L. infantum* is not exceptional. A multicentre study including 2067 cats from six Mediterranean countries reported an overall seropositivity of 17.3%. Shelter or free-roaming conditions and feline immunodeficiency virus infection were identified as risk factors, while seropositivity was associated with weight loss, lymphadenomegaly, gingivostomatitis, oral lesions, hyperglobulinaemia and a reduced albumin-to-globulin ratio (Carbonara et al., 2024).

Current diagnostic approaches include quantitative serology, molecular assays, cytology, histopathology, and immunohistochemistry. No single method provides complete diagnostic certainty, and complementary investigations are recommended when serology is negative or weakly positive in clinically suspect cats (Pennisi et al., 2026). Many of these techniques are difficult to integrate into immediate first-opinion decision-making. Molecular methods, western blot (WB), immunohistochemistry and parasite culture require adapted laboratory facilities, trained personnel or specific instrumentation. Cytology and histopathology depend on appropriate lesion selection and may require invasive sampling, whereas indirect fluorescent antibody testing (IFAT) and enzyme-linked immunosorbent assays (ELISA) generally do not provide results during the consultation. These limitations may delay recognition of FeL when clinical abnormalities are mild or non-specific. This contrasts with canine leishmaniosis (CanL), in which rapid immunochromatographic tests (ICTs) are widely incorporated into routine diagnostic workflows (Villanueva-Saz et al., 2022). The relevant question is therefore not simply why rapid tests are widely used in dogs but less frequently in cats. Rather, it is whether the canine serological paradigm is appropriate for feline infection.

## 2 Why cats require species-adapted serology

Cats and dogs exposed to *L. infantum* do not necessarily develop diagnostically equivalent antibody responses. In naturally exposed cats and dogs from the same endemic area, similar overall frequencies of parasite exposure or infection were observed when serological, molecular and cell-mediated immune markers were considered collectively. However, dogs exhibited significantly higher anti-*L. infantum* antibody levels than cats, indicating host-dependent differences in the immune response (Priolo et al., 2022).

In feline monocyte-derived macrophages infected *in vitro* with *L. infantum*, interleukin (IL)-2, interferon (IFN)-γ and tumour necrosis factor (TNF)-α were upregulated, whereas IL-4 and IL-6 remained relatively stable and IL-10 expression decreased. This predominantly T helper 1-like inflammatory profile may contribute to partial control of infection and has been proposed as one possible explanation for the generally milder clinical course observed in cats compared with dogs (Louzada-Flores et al., 2026).

Early evidence also supports a species-specific immunological response to *Leishmania* spp. infection in cats, including parasite-specific cell-mediated response and host-parasite interactions that may differ from those described in dogs and humans (Pereira and Maia, 2021). These observations suggest that the feline immune response to *L. infantum* should not be extrapolated from the canine model (Pereira and Maia, 2021; Louzada-Flores et al., 2026).

Different diagnostic measures have been described when serological techniques have been evaluated in FeL. In this sense, Bayesian analysis performed without assuming a perfect diagnostic gold standard estimated median sensitivities of 97% for WB, 75% for IFAT and 70% for ELISA, with corresponding specificities of 99%, 97% and 98%, respectively (Persichetti et al., 2017). In apparently healthy stray cats from Zaragoza, Spain, positivity ranged from 2.2% by IFAT and 2.8% by ELISA to 14.5% by WB, whereas *L. infantum* DNA was detected in blood by quantitative PCR in 5.6% of cats. Overall, 15.6% were considered infected based on serological and/or molecular evidence, but only two animals were positive by all methods (Alcover et al., 2021). Although these results cannot be directly extrapolated to cats with overt FeL, they illustrate how recognition of feline infection depends on the diagnostic platform selected.

Further evidence of this diagnostic heterogeneity comes from a study of 166 shelter cats, in which markedly different detection rates were obtained by ELISA (15%), IFAT (53.6%), molecular methods (3.6%) and parasitological examination (1.8%) (Silva et al., 2023). Although these estimates depend on the population studied and the diagnostic criteria and cut-offs applied, the marked differences between methods further demonstrate that individual assays are not interchangeable and support the use of complementary diagnostic approaches (Silva et al., 2023).

Anti-*Leishmania* antibody detection should therefore not be considered directly equivalent across host species. Within feline serology, antigen selection influences antibody recognition, as different *L. infantum* antigens generate heterogeneous and partly complementary serological profiles (Lima et al., 2026). Assay detection chemistry can likewise affect analytical performance in feline samples, as evidenced by the improved sensitivity and test-line intensity achieved with a latex-based lateral-flow format compared with a conventional colloidal-gold system (Puerta López et al., 2026). Immunoglobulin-binding reagents may also exhibit species-dependent affinities across mammalian taxa (Villanueva-Saz et al., 2026). Nevertheless, antigen selection, detection chemistry and detection thresholds should primarily be regarded as variables requiring feline-specific optimisation and validation rather than as established intrinsic differences between feline and canine serology. Serological specificity may be further affected by exposure to other *Leishmania* species. For example, IFAT cross-reactivity between *L. infantum* and *Leishmania tarentolae* in naturally exposed outdoor cats represents an additional diagnostic challenge and demonstrates the importance of antigen selection and assay-specific validation in feline serology (Mendoza-Roldan et al., 2025).

## 3 What point-of-care serology could add

The primary value of a point-of-care assay lies not in replacing conventional laboratory diagnostics, but in providing rapid, clinically relevant information at the time of decision-making as an initial step in a diagnostic algorithm. Although this approach is routinely used in dogs (Maia and Campino, 2018), an externally validated, feline-adapted point-of-care serological strategy has not yet become established in clinical practice (Pennisi et al., 2026). An ICT with insufficient sensitivity for low-level feline antibody responses could reinforce the perception that FeL is rare by generating negative results in animals in which clinical suspicion is already low. By contrast, a feline-adapted assay could lower the threshold for further investigation and alter clinical reasoning at presentation.

A recent clinical case report supports this potential. In a cat with unilateral uveitis, a commercial rapid test for anti-*L. infantum* antibodies was positive in serum and aqueous humour. Infection was subsequently supported by ELISA, WB, PCR in blood and aqueous humour, and parasite isolation with molecular identification as *L. infantum* (González et al., 2026). The ICT did not independently establish the diagnosis; instead, immediate serological information was incorporated into a broader diagnostic investigation.

In an evaluation of a commercial ICT involving 186 serum samples from domestic and non-domestic species, complete agreement between the ICT and reference serological classification was observed among the feline samples included. Marked species-dependent differences occurred in other taxa, particularly herbivores, highlighting the importance of conjugate-immunoglobulin interactions and species-specific assay validation (Villanueva-Saz et al., 2026). More recently, development of a feline-specific latex-based ICT demonstrated that optimisation of detection chemistry can substantially improve serological detection in cats. Compared with a conventional colloidal-gold system, the latex-based assay showed markedly higher sensitivity while maintaining very high specificity and improving test-line signal intensity (Puerta López et al., 2026).

## 4 The appropriate clinical role of a feline ICT

A feline-specific ICT should not be viewed as a stand-alone test capable of diagnosing clinical FeL. A positive antibody result does not independently establish that *L. infantum* is responsible for clinical abnormalities, because exposure or subclinical infection may coexist with unrelated disorders. Similarly, cats with compatible clinical signs and negative or weakly positive serology may still require molecular or parasitological investigation (Pennisi et al., 2026). The greatest value of a validated feline ICT may therefore be as a first-line triage tool within a multimodal diagnostic pathway, especially in cats from *L. infantum*-endemic areas presenting with compatible clinical signs and/or compatible laboratory abnormalities. An immediate positive result could strengthen clinical suspicion and justify further testing, including quantitative serology, molecular testing, cytology, or direct parasite detection such as immunohistochemistry or parasite isolation. Conversely, a negative result should not terminate the diagnostic investigation when clinical suspicion remains high, and the use of additional confirmatory techniques should be recommended. Diagnostic usefulness depends not only on sensitivity and specificity but also on the target population and the clinical decisions triggered by the result. Point-of-care testing may therefore provide value if it accelerates recognition of an underdiagnosed differential and directs appropriate confirmatory investigation.

Beyond its potential use in clinically suspected cases, point-of-care serology may also have a role in selected apparently healthy cats with epidemiological exposure to *L. infantum*, particularly those living in or travelling to endemic areas. In this setting, an ICT should be regarded as an initial screening step rather than a definitive diagnostic procedure, with positive or otherwise clinically relevant findings prompting quantitative serology and, when indicated, additional laboratory or molecular investigation. A possible framework for this approach is proposed in Fig. 1.

**Fig. 1:**
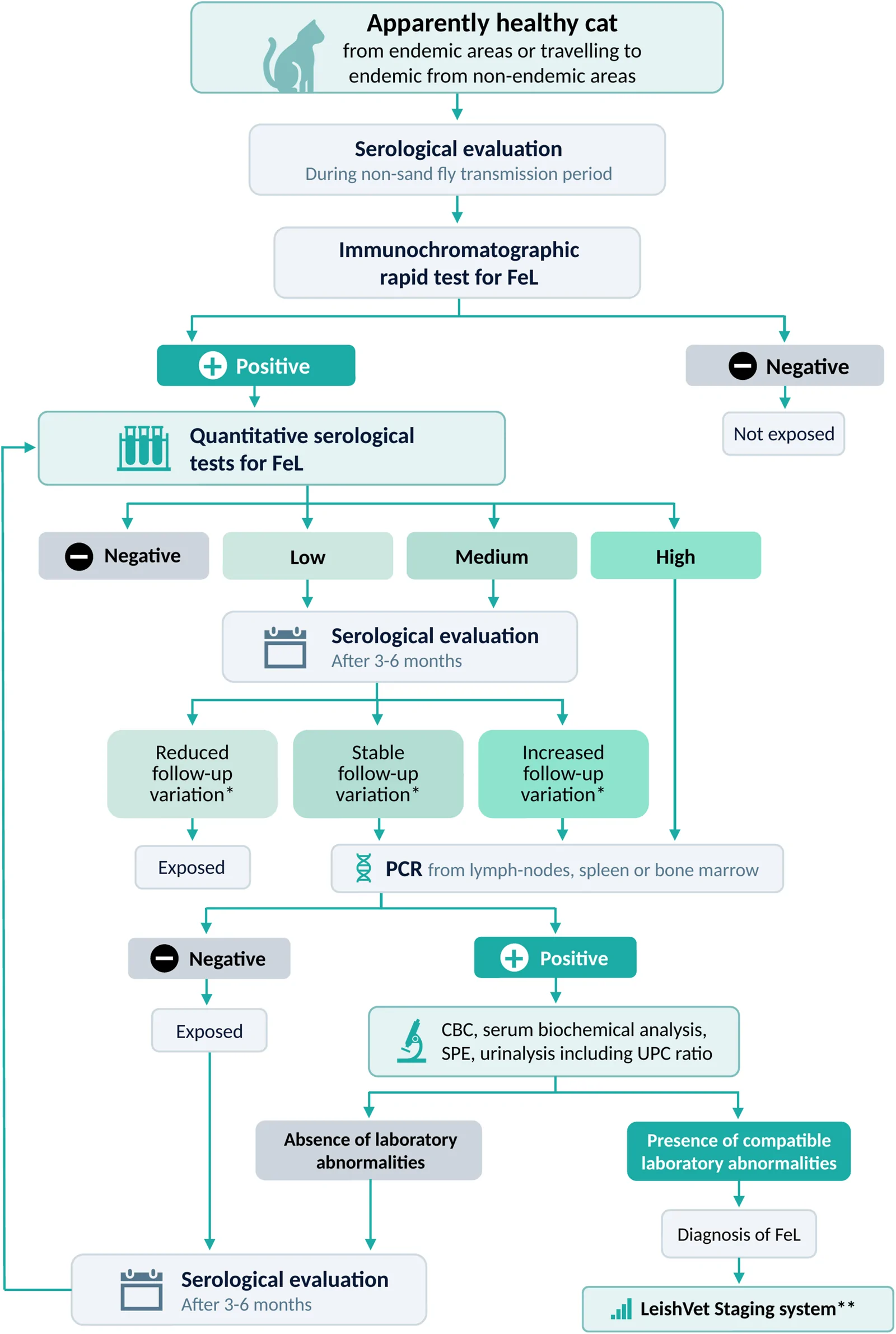
Flowchart for the serological screening and follow-up of clinically healthy cats for *Leishmania infantum*. *Serological variation was defined as an increase or decrease of at least two dilution steps in indirect fluorescent antibody test titre, or as a relative increase or decrease of > 40% in enzyme-linked immunosorbent assay antibody levels compared with the previous measurement. **Please refer to the LeishVet guidelines for treatment recommendations (Pennisi et al., 2026). *Abbreviations*: CBC, complete blood count; FeL, feline leishmaniosis; PCR, polymerase chain reaction; SPE, serum protein electrophoresis; UPC, urinary protein-to-creatinine.

## 5 How should feline point-of-care tests be validated?

Future feline ICTs should be assessed prospectively in well-characterised populations that include cats with confirmed clinical FeL, infected but apparently healthy cats, animals with other infectious, inflammatory or neoplastic diseases, and unexposed controls. Particular attention should be given to spectrum bias, cross-reactivity, reproducibility of visual interpretation and performance in cats with low antibody concentrations. Comparative assessment using serum, plasma and whole blood would also clarify applicability under genuine point-of-care conditions (Puerta López et al., 2026).

A major methodological problem is the absence of a perfect diagnostic gold standard for leishmaniosis. In CanL, this limitation can be partially mitigated by the availability of several serological methods validated specifically in dogs (Miró et al., 2008). The corresponding range of assays validated for FeL remains considerably more limited. Estimating sensitivity and specificity of a new ICT solely against another imperfect serological test may consequently lead to misclassification of discordant samples and biased estimates of diagnostic accuracy. Composite reference standards incorporating molecular, parasitological and serological evidence, or alternatively latent-class approaches that do not assume the existence of a perfect reference test, may provide more robust estimates of diagnostic performance. Multicentre studies would also be desirable to assess reproducibility across laboratories (Mahachi et al., 2024), geographical and clinical populations (Solano-Gallego et al., 2014), diagnostic purposes and disease presentations (Villanueva-Saz et al., 2022).

## 6 A distinct diagnostic priority

The updated LeishVet recommendations identify the lack of externally validated commercial serological assays for feline *Leishmania* infection as a persisting diagnostic limitation (Pennisi et al., 2026). Concurrently, studies published in 2026 suggest that this limitation is beginning to be addressed through feline-focused evaluation and optimisation of lateral-flow technologies (González et al., 2026; Puerta López et al., 2026; Villanueva-Saz et al., 2026).

Several studies indicate that feline *L. infantum* infection is epidemiologically measurable, with patterns in cats differing from those reported in dogs, and that diagnostic outcomes vary according to the method employed (Pennisi et al., 2015; Persichetti et al., 2017; Alcover et al., 2021; Priolo et al., 2022). Immunological findings provide an additional mechanistic perspective on these host-specific differences (Louzada-Flores et al., 2026), while advances in lateral-flow technology demonstrate the feasibility of optimising assay chemistry for feline samples (Puerta López et al., 2026).

Taken together, these findings support prioritising the development, optimisation and external validation of serological tools specifically evaluated in cats, rather than considering the absence of a feline-adapted ICT as the principal diagnostic gap. Further work should characterise analytical and clinical performance in well-defined feline populations, assess how test performance varies with clinical status and antibody response, and determine the settings in which point-of-care serology provides meaningful additional value. Prospective clinical investigations will also be required to establish whether feline-adapted ICTs improve recognition of *L. infantum* infection, support appropriate selection for confirmatory testing, or contribute to earlier identification of clinically relevant cases.

## 7 Conclusions

FeL requires a diagnostic approach that reflects the biological and serological characteristics of cats rather than relying on direct extrapolation from canine models. For this purpose, species-adapted point-of-care serology could improve early recognition of suspected cases and support more timely selection of confirmatory tests, provided that these assays are rigorously validated and interpreted within a multimodal diagnostic framework. Their development should therefore be considered an important step towards improving both the clinical diagnosis and epidemiological understanding of *L. infantum* infection in cats.

## Ethical approval

Not applicable.

## Credit authorship contribution statement

**Sergio Villanueva-Saz:** Conceptualization, Writing - original draft, Writing-review & editing, Resources, Supervision. **Diana Marteles-Aragüés:** Conceptualization, Writing - original draft, Writing-review & editing, Resources, Supervision.

## Data availability

The data supporting the findings of this study are included in this published article.

## Statement on the use of AI-assisted technologies

No generative AI or AI-assisted tools were used in the creation or modification of figures, images, or artwork in this manuscript. All graphical materials were produced by professional staff graphic designers using conventional design software.

## Funding

The authors received no financial support for the research, authorship, and/or publication of this article.

## Declaration of competing interests

The authors declare that they have no known competing financial interests or personal relationships that could have appeared to influence the work reported in this paper.
